# Comparative Analysis and Expression Patterns of the *PLP_deC* Genes in *Dendrobium officinale*

**DOI:** 10.3390/ijms21010054

**Published:** 2019-12-20

**Authors:** Lei Zhang, Chunyan Jiao, Yunpeng Cao, Xi Cheng, Jian Wang, Qing Jin, Yongping Cai

**Affiliations:** 1 School of Life Sciences, Anhui Agricultural University, Hefei 230036, China; zhanglei123@ahau.edu.cn (L.Z.); 15212426671@163.com (C.J.); chengxi90@ahau.edu.cn (X.C.);; 2 Anhui Provincial Engineering Technology Reserach Center for Development & Utilization of Regional Characteristic Plants, Anhui Agricultural University, No. 130, Changjiang West Road, Hefei 230036, China; 3 Key Laboratory of Cultivation and Protection for Non-Wood Forest Trees, Ministry of Education, Central South University of Forestry and Technology, Changsha 410004, China; xfcypeng@126.com; 4 Key Lab of Non-wood Forest Products of State Forestry Administration, College of Forestry, Central South University of Forestry and Technology, Changsha 410004, China

**Keywords:** *Dendrobium officinale*, *PLP_deC*, bioinformatics, expression pattern analysis, evolution

## Abstract

Studies have shown that the type II pyridoxal phosphate-dependent decarboxylase (*PLP_deC*) genes produce secondary metabolites and flavor volatiles in plants, and TDC (tryptophan decarboxylase), a member of the *PLP_deC* family, plays an important role in the biosynthesis of terpenoid indole alkaloids (TIAs). In this study, we identified eight *PLP_deC* genes in *Dendrobium officinale* (*D. officinale*) and six in *Phalaenopsis equestris* (*P. equestris)*, and their structures, physicochemical properties, response elements, evolutionary relationships, and expression patterns were preliminarily predicted and analyzed. The results showed that *PLP_deC* genes play important roles in *D. officinale* and respond to different exogenous hormone treatments; additionally, the results support the selection of appropriate candidates for further functional characterization of *PLP_deC* genes in *D. officinale*.

## 1. Introduction

*Dendrobium officinale* Kimura et Migo (also known as *D. catenatum*) is a perennial herb that is commonly used as a valuable Chinese herbal medicine and has a long evolutionary history among orchids. *D. officinale* is rich in alkaloids [1,2], and its genome, transcriptome, and metabolome indicate that *D. officinale* may also contain terpenoid indole alkaloids (TIAs) [3,4,5]. The common precursor of TIAs is strictosidine, which is formed by the combination of tryptamine and secologanin [6,7]. Tryptophan decarboxylase (TDC), which catalyses the formation of tryptamine, belongs to the type II pyridoxal phosphate-dependent decarboxylase (PLP_deC) family [8]. To date, the actual roles of many PLP_deCs in plants are still unknown due to a lack of relevant protein sequences and information about the biochemical properties. In particular, the role of PLP_deC in the alkaloid synthesis pathway of *D. officinale* has not been reported.

Pyridoxal 5′-phosphate (PLP) is the active form of vitamin B6 and is used by a variety of enzymes in all organisms [8]. Previously, we classified all PLP-dependent enzymes into at least five structural groups based on their protein structures [8,9]. Among them, the type I group is the most common and contains aminotransferases, decarboxylases, and an enzyme that catalyzes α-, β-, or γ-eliminations. Type II encodes the enzymes involved in β-elimination reactions. Type III is primarily alanine-racemase specific, while type IV enzymes typically include D-alanine aminotransferases. Type V enzymes are the most diverse, including glycogen and starch phosphorylases. One important group of PLP-dependent enzymes belongs to the PLP_deC family, which includes aromatic-L-amino acid decarboxylase (AAD), glutamic acid decarboxylase (GAD), and histidine decarboxylase (HDC) [10]. The biological functions of plant and animal AADs are closely related to their corresponding substrate selectivity and catalytic reactions; thus, some AADs, such as tyrosine decarboxylase (TYDC) and tryptophan decarboxylase (TDC), are further annotated based on their principal substrates [8,11]. These enzymes catalyze the decarboxylation of aromatic L-amino acids and are primarily involved in the synthesis of secondary metabolites in plants [12,13].

Numerous data indicate that *PLP_deC* exhibits tissue-specific and inducible transcript accumulation during plant development. In addition, several roles of *PLP_deC* in plant development have been identified. For example, TDC is a key enzyme that links primary and secondary metabolism with high substrate specificity [14,15]. In addition, the transcript levels of *PLP_deC* genes are affected by abscisic acid (ABA), methyl jasmonate (MeJA), salicylic acid (SA), and abiotic stress [16,17].

In 2015, Chinese scientists announced that they had completed the genome sequence of the orchid *Phalaenopsis equestris* [18]. *D. officinale* and *P. equestris* (Schauer) Rchb.f. are epiphytes in the family Orchidaceae. The draft of the *D. officinale* genome sequence was reported recently [4,19]. To further understand the *PLP_deC* gene family in orchids, we identified 8 and 6 *PLP_deC* genes from the genomes of *D. officinale* and *P. equestris,* respectively, and analyzed their phylogenetic relationships, gene structures, cis-regulatory elements, tissue expression patterns, and expression profiles under MeJA, ABA, and SA treatments. Our results may not only improve the current understanding of the evolutionary expansion, sequence conservation, and functional differentiation of *PLP_deC* genes but also provide in-depth basic biological information for further studies of the evolution of these genes in Orchidaceae.

## 2. Results

### 2.1. Sequence Analysis of *PLP_deC* Family Members

HMMER software was used to identify candidate genes in the genomes of *D. officinale* and *P. equestris*. All candidate genes were submitted to Pfam and SMART for verifying the presence of the PLP_deC domain. The sequences without the conserved domain and the redundant sequences were removed. Finally, a total of 14 *PLP_deC*-family sequences were obtained in *D. officinale* and *P. equestris*. The basic information of each *PLP_deC* was listed in Table 1. We found that in *D. officinale*, the molecular weights ranged from 34.48 kDa (DoGAD4) to 79.29 kDa (DoAAD2), with an average molecular weight of 57.64 kDa, and the theoretical isoelectric points ranged from 5.27 (DoHDC1) to 7.52 (DoAAD1), with an average value of 6.12. In *P. equestris*, the molecular weights ranged from 53.44 kDa (PeHDC1) to 57.26 kDa (PeGAD3), with an average molecular weight of 55.53 kDa, while the theoretical isoelectric points ranged from 5.53 (PeGAD1) to 8.55 (PeGAD3), with an average of 6.52. These results showed that most genes have an acidic pI. We analyzed the subcellular localization of 8 and 6 PLP_deC-family protein sequences in *D. officinale* and *P. equestris* by Target P 1.1. and the results showed the presence of a signal peptide in DoAAD1 and DoAAD3, suggesting their cellular localization in extracellular with a probability of 0.735 and 0.561, The proteins of DoHDC1, PeAAD1, PeAAD2 and PeHDC1 are possibly located in other cellular compartments, e.g. chloroplast transit peptide and mitochondrial, whereas the location of the remaining eight proteins is unknown (Table 2).

### 2.2. Analysis of the Gene Structures, Conserved Motifs, and Phylogenetic Relationships of *PLP_deC* Genes

**Figure 1:**
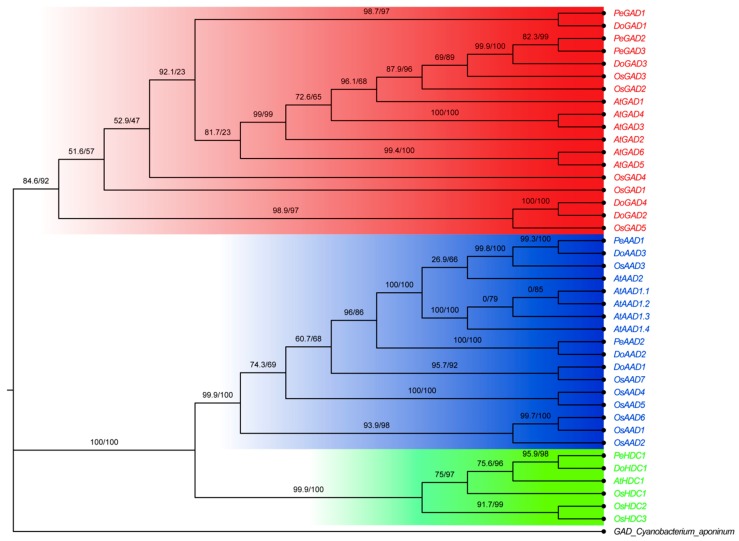
Phylogenetic analysis of *PLP_deC* genes from *Dendrobium officinale*, *Phalaenopsis equestris*, *Oryza sativa*, and *Arabidopsis thaliana*. The maximum likelihood (ML) tree was created using IQ-TREE with 8 *D. officinale* (Do), 6 *P. equestris* (Pe), 15 *O. sativa* (Os) and 12 *A. thaliana* (At) PLP_deC protein sequences. The red, green, and blue colors indicate GADs, HDCs, and AADs, respectively. Bootstrap supports are indicated at each branch.

To clarify the evolutionary relationships among the *PLP_deC* genes, we compared the PLP_deC proteins from *Arabidopsis thaliana (A. thaliana)*, *Oryza sativa (O. sativa)*, *D. officinale* and *P. equestris*. We used the maximum likelihood (ML) method to construct a phylogenetic tree using IQ-TREE software. As shown in Figure 1, the 42 *PLP_deC* genes could be divided into three subfamilies: GAD, HDC, and aromatic-L-AAD. The *PLP_deC* genes in *D. officinale* and *P. equestris* were named according to their relative homology with *A. thaliana* and *O. sativa* genes. Among them, the GAD subfamily was the largest, with 18 *PLP_deC* genes, and the HDC subfamily was the smallest, with 6 members.

**Figure 2:**
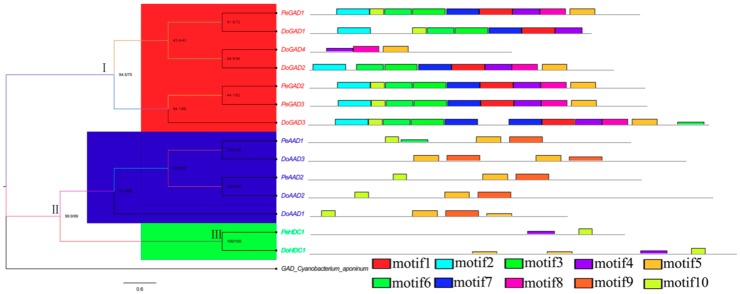
Distribution of 10 putative conserved motifs in *PLP_deC* proteins.

To further analyze the gene structures and conserved motifs of the *PLP_deC* family members in *D. officinale* and *P. equestris*, a total of 10 motifs were identified from the amino acid sequences of the *PLP_deC* family members using the Multiple EM for Motif Elicitation (MEME) software (Figure 2). We checked these motifs to verify they are known domains by pfam and the motif logos were generated using online MEME program (Figure S1). Group I, which includes GAD sequences, harbors all the motifs; group II, which includes AAD sequences, harbors motifs 5, 6, 9, and 10; and group III, which includes HDC sequences, harbors motifs 4, 5, and 10. These results show that most *PLP_deC* genes in the same subfamily have highly similar motifs, which supports their close evolutionary relationships and the reliability of the constructed phylogenetic tree. Remarkably, motif 10 and motif 5 are present in all subfamilies, but they are not part of the PLP_deC domain. Thus, we speculate that they may perform other specific functions. In addition, we used the online Gene Structure Display Server to analyze the gene structures. The results showed that the number of exons in *PLP_deC* genes ranged from 2 to 14. For example, there are 3–8 exons in subfamily GAD, 2–14 exons in subfamily AAD, and 5 or 7 exons in subfamily HDC. These results indicate that the number of exons in the *PLP_deC* gene family has increased or decreased during evolution, providing a basis for functional differences among the homologous *PLP_deC* genes (Figure S2).

### 2.3. Analysis of Evolutionary Patterns of *PLP_deC* Genes

**Figure 3:**
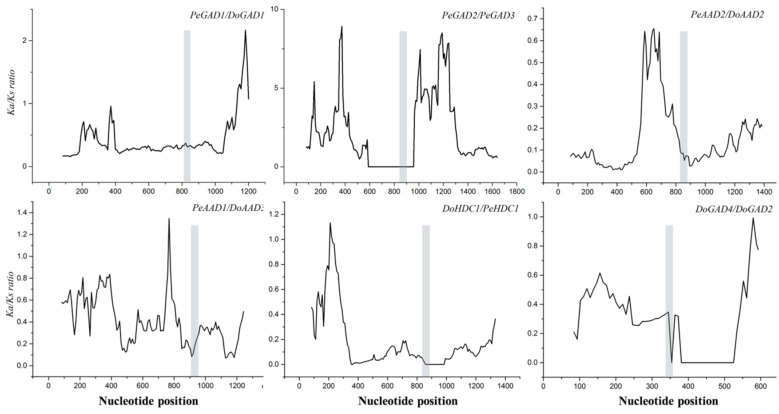
Sliding window analysis of Ka/Ks for each gene pair. The window size is 150 bp, and the step size is 9 bp. The grey region represents the conserved domain.

We analyzed the relationships of *D. officinale* and *P. equestris* homologous gene pairs to further analyze the evolutionary patterns of PLP_deC genes. We obtained six homologous gene pairs (*PeGAD1*–*DoGAD1*, *DoGAD2*–*DoGAD4*, *PeAAD1*–*DoAAD3*, *DoAAD2*–*PeAAD2*, *PeHDC1*–*DoHDC1*, and *PeGAD2*–*PeGAD3*) and calculated their Ka, Ks, and Ka/Ks values. The estimation of the synonymous (Ks) and nonsynonymous (Ka) nucleotide substitution rates is one of the important parameters for molecular evolutionary analyses, which are, respectively, defined as the number of synonymous substitutions per synonymous site and the number of nonsynonymous substitutions per nonsynonymous site per year or per generation. It is generally recognized that Ka/Ks > 1, Ka/Ks = 1, and Ka/Ks < 1 indicate positive, neutral, and purifying selection, respectively [20]. The experimental results showed that the Ka/Ks values of two homologous pairs (*DoAAD2*–*PeAAD2*, *PeHDC1*–*DoHDC1*) were lower than 0.3, the Ka/Ks values of the three homologous pairs (*PeAAD1*–*DoAAD3, DoGAD2*–*DoGAD4,* and *PeGAD1*–*DoGAD1*) were between 0.3 and 1, and the Ka/Ks value of one of the gene pairs (*PeGAD2*–*PeGAD3*) was greater than 1 (Table 3). These data indicated that most homologous *PLP_deC* gene pairs are subjected to purifying selection. To further understand the influence of selection on the homologous gene pairs, we performed a sliding window analysis. The grey regions shown in Figure 3 represent the conserved domains. The Ka/Ks values for the conserved domains were typically less than 1, indicating that these pairs have purifying selection. These findings suggest that purifying selection may have played a key role in the evolution of this gene family.

### 2.4. *D. officinale PLP_deC* Gene Expression in Different Tissues

**Figure 4:**
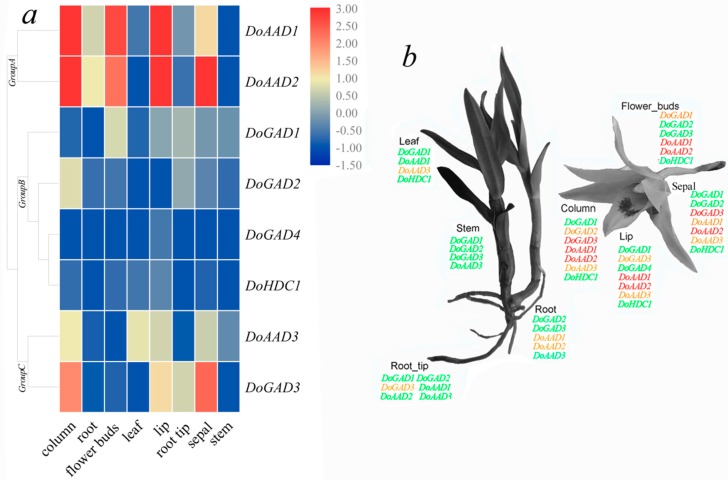
Expression profiles of *PLP_deC* genes in different tissues. (**a**) Heatmap of the in silico expression analysis in different tissues and organs. Blue and red indicate lower and higher transcript abundance, respectively. (**b**) *PLP_deC* genes expressed in different tissues and organs. Green, yellow, and red indicate low (0.01–0.71 FPKM), medium (1–1.71 FPKM), and high (3.42–28.4 FPKM) expression, respectively.

To understand the gene expression pattern of the *PLP_deC* genes in *D. officinale*, we performed an overall in silico analysis of gene expression profiles in eight tissues (root, root tip, stem, leaf, sepal, column, lip, and flower bud). These *PLP_deC* genes exhibited distinct organ-specific expression and could be divided into three groups. As shown in Figure 4, in group A, two genes (*DoAAD1* and *DoAAD2*) were highly expressed in the flower bud and lip columns, indicating that these *PLP_deC* genes may be involved in the development of these tissues. In addition, *DoAAD2* had a high expression level in the sepal, indicating that *DoAAD2* may be involved in sepal development. The four *PLP_deC* genes in group B were generally expressed in low amounts in all eight tissues. Remarkably, a homologous gene pair (*DoGAD2*–*DoGAD4*) exhibited similar patterns of expression and had relatively low transcript abundance in different tissues. In group C, *DoAAD3* had low expression in all tissues. *DoGAD3* had higher expression levels in the column and sepal. Overall, the *PLP_deC* genes with high expression levels may be involved in tissue growth and differentiation in *D. officinale*.

### 2.5. Identification of Cis-Acting Elements of *PLP_deC* Genes

**Figure 5:**
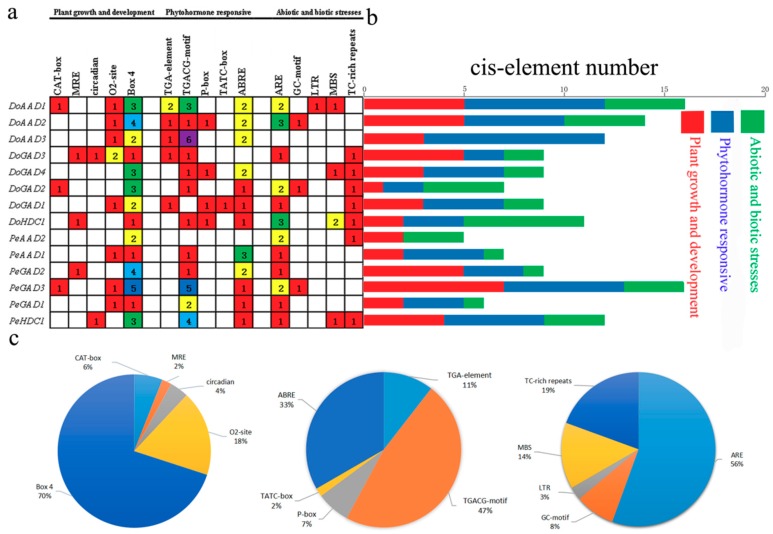
Numbers of cis-acting elements in all the *PLP_deC* genes of *D. officinale* and *P. equestris*. (**a**) The different colors and numbers in the grid indicate the numbers of different promoter elements in each *PLP_deC* gene. (**b**) The different colored histogram represents the numbers of cis-acting elements in the different categories. The red, blue, and green indicate plant growth and devolepment, phytohormone response, and biotic and abiotic stress, respectively. (**c**) The pie charts indicate the percentages of different promoter elements in the different categories.

Plant growth and development are regulated by different cis-elements in genes. Therefore, we used the PlantCARE database to identify and analyze cis-elements in the *PLP_deC* genes and identified three categories of cis-elements: plant growth and development, phytohormone response, and biotic and abiotic stress response. The cis-acting elements in the growth and development category included the CAT-box for meristem expression, O_2_-site for zein metabolic regulation, MRE and Box 4 for light response, and others. We identified 35 Box-4 motifs, which compose the largest portion of the growth and development category. All the *PLP_deC* genes contained Box-4 elements (Figure 5a), indicating that the expression of all these genes is closely related to light. In the phytohormone response category, TCA elements for SA response and P-boxes and TATC-boxes for gibberellic acid response were identified. Notably, TGACG motifs for MeJA response accounted for 47% of the phytohormone response category (Figure 5c), while ABRE elements for ABA response accounted for 33%. The last category was biotic and abiotic-stress-response elements, including AREs and GC motifs for anerobic response, TC-rich repeats for defence and stress response, and MBSs and LTRs for low temperature response. Our data suggested that the *PLP_deC* genes may respond to abiotic stresses in *D. officinale*.

### 2.6. Analysis of the Expression Patterns of *PLP_deC* Genes in *D. officinale*

In plants, many stress responses are modulated or mediated by various signaling pathways that are inseparable from gene expression and regulation [21]. To investigate the responses of the *PLP_deC* genes to different hormone treatments, we used qRT-PCR to analyze their expression under MeJA, ABA, and SA treatments.

**Figure 6:**
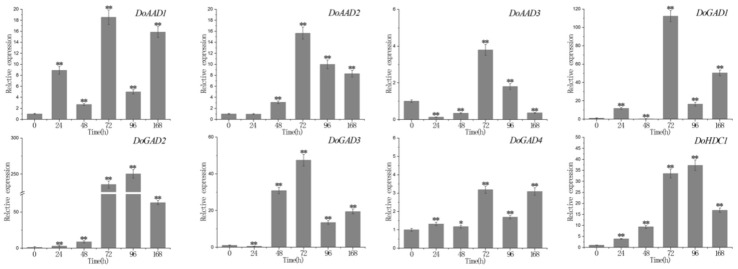
The expression levels of *PLP_deC* genes in *D. officinale* under abscisic acid (ABA) treatment. The x-axis represents the treatment time, and the y-axis represents the gene expression level. Error bars indicate the mean and standard deviation (SD). The asterisks indicate significant difference relative to the time 0. ** significant difference (*p* < 0.01), * significant difference (*p* < 0.05).

**Figure 7:**
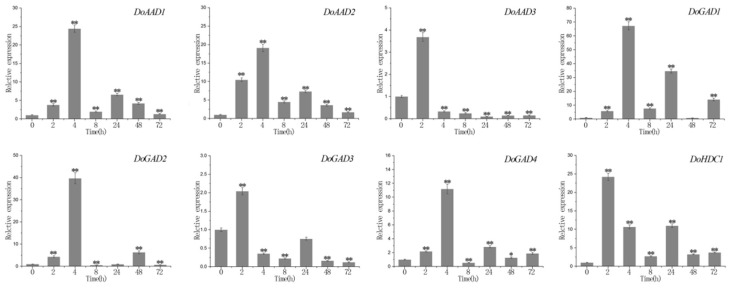
The expression level of *PLP_deC* genes in *D. officinale* under methyl jasmonate (MeJA) treatment stress. The x-axis represents the treatment time, and the y-axis represents the gene expression level. Error bars indicate the mean and standard deviation (SD). The asterisks indicate significant difference relative to the time 0. ** significant difference (*p* < 0.01), * significant difference (*p* < 0.05).

**Figure 8:**
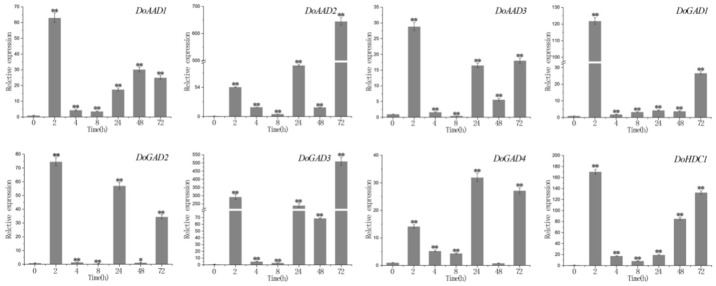
The expression levels of *PLP_deC* genes in *D. officinale* under salicylic acid (SA) treatment. The x-axis represents the treatment time, and the y-axis represents the gene expression level. Error bars indicate the mean and standard deviation (SD). The asterisks indicate significant difference relative to the time 0. ** significant difference (*p* < 0.01), * significant difference (*p* < 0.05).

In the ABA treatment, we found that *DoAAD1*, *DoAAD2*, *DoAAD3*, *DoGAD1*, *DoGAD3*, and *DoGAD4* reached their highest expression levels after 72 hours, and *DoGAD1* and *DoGAD3* were strongly upregulated (by more than 110-fold and 50-fold, respectively). The expression of two *PLP_deC* genes (*DoGAD2* and *DoHDC1*) peaked at 96 h, and *DoGAD2* was strongly upregulated (more than 250-fold) (Figure 6). In the MeJA treatment, three *PLP_deC* genes *(DoAAD3*, *DoGAD3*, and *DoHDC1)* showed strong upregulation at 2 h (Figure 7), while five PLP_deC genes (*DoAAD1*, *DoAAD2*, *DoGAD1*, *DoGAD2*, and *DoGAD4*) were strongly upregulated after 4 h of treatment. In the SA treatment, the expression levels of *DoAAD1*, *DoAAD3*, *DoGAD1*, *DoGAD2*, and *DoHDC1* were strongly upregulated at 2 h (Figure 8). However, *DoAAD2* and *DoGAD3* were strongly upregulated after treated with SA for 72 h of treatment (over 645- and 508-fold, respectively).

## 3. Discussion

The type II PLP_deC enzymes are an important group of carboxylases among the PLP-dependent enzymes. Many data indicate that PLP_deCs show developmental, tissue-specific, and inducible transcript accumulation during plant development [19,22]. In this paper, we identified 8 and 6 *PLP_deC* genes from the *D. officinale* and *P. equestris* genomes, respectively. According to the phylogenetic analysis, all the *PLP_deC* genes from *A. thaliana*, *O. sativa*, *D. officinale*, and *P. equestris* were clustered into GAD, AAD, and HDC subclasses based on their high sequence similarity, which is consistent with the ML tree of *PLP_deC* genes from the genomes of 18 species and previously published articles [17]. However, some of these genes might have evolved with different functions. For example, in tomato, *SlHDC19* and *SlHDC6* do not act on histidine but prefer tyrosine as their substrate [17]. Furthermore, many HDCs are biased toward serine rather than histidine based on biochemical analysis [18]. Therefore, although their sequences have high similarity, *PLP_deC* genes have individual substrate specificities; we should perform an in-depth biochemical characterization to understand their precise functions [23].

Gene duplication is a common phenomenon in species and contributes to the generation of biodiversity during evolution [24]. To date, the chromosome assemblies of the *D. officinale* and *P. equestris* genomes have not yet been finished [25], and thus, the homologous genes of *D. officinale* and *P. equestris* cannot yet be clearly shown on the chromosomes. Therefore, we are unable to determine the type of replication events that have occurred between these species. To further understand the evolutionary patterns of the *PLP_deC* genes, we calculated the Ka and Ks values of homologous gene pairs. We predicted that two gene pairs (*PeGAD1–DoGAD1* and *PeAAD2–AAD2*) are evolved from the genome-wide duplication events shared by *D. officinale* and *P. equestris*, because their values of Ks are 0.7 to 1.1 [26]. The Ka/Ks values in this experiment were less than 1 for all the homologous gene pairs except for *PeGAD2–PeGAD3*, implying that these gene pairs have undergone purifying selection during evolution. In addition, we noticed four homologous gene pairs (*PeGAD1–DoGAD1*, *PeAAD2–AAD2*, *PeAAD1–DoAAD3*, and *PeHDC1–DoHDC1*) had the comparatively high Ka/Ks values (>0.5), showing that these gene pairs have undergone rapid evolutionary diversification after duplication events in the course of evolution [24].

Analysis of *D. officinale PLP_deC* gene expression in different tissues can help us better understand the tissue specificity of the *PLP_deC* genes. Therefore, expression profiles for all the *PLP_deC* genes were established using published RNA-sequence data. Among them, *DoAAD1*, *DoAAD2*, and *DoGAD3* were highly expressed in different tissues, indicating that these *PLP_deC* genes play important roles during *D. officinale* growth and development. For example, GADs are involved in many cellular processes, including pollen-tube development in *Arabidopsis* and *Picea wilsonii* [25,27]. In this study, some cis-acting elements associated with particular tissues were identified in the *PLP_deC* gene promoter regions, such as the O_2_-site required for seed expression and the CAT-box required for meristem organization. The corresponding *PLP_deC* genes (such as *DoAAD1* and *DoAAD2*) might play important role in the formation of reproductive organs.

Many studies have suggested that the expression levels of *PLP_deC* genes are also influenced by abiotic and biotic stresses [28,29,30]. Furthermore, plant hormones such as ABA, SA, and ethylene also modulate the expression of these genes [17,29,30]. In this study, we identified a number of cis-acting elements in the promoter regions of *PLP_deC* genes in both *D. officinale* and *P. equestris*, such as MBS, MRB, Box 4, and ABRE. We found that these *PLP_deC* genes contain at least one abiotic stress cis-element, which showed that they may contribute to biotic and abiotic stress responses. To further investigate the responses of the *PLP_deC* genes to different hormones, we analyzed their expression with the treatments of MeJA, ABA, and SA by qRT-PCR. We observed that the *PLP_deC* genes had significantly differential expression patterns under different treatments. Some of the *PLP_deC* genes showed strong upregulation under the treatments, indicating that these genes play key roles in the abiotic stress responses of *D. officinale*. For example, *DoAAD2* was strongly upregulated (645-fold) after 72 h of SA treatment. Overall, we found that the *PLP_deC* genes of *D. officinale* responded to abiotic stress, such as MeJA, ABA, and SA stresses. These results provide strong evidence that the *PLP_deC* genes in plants are involved in abiotic stress responses.

## 4. Materials and Methods

### 4.1. Materials and Treatments

Seedlings of *D. officinale* were planted on Murashige and Skoog (MS) medium and placed in a tissue culture chamber at a constant temperature of 25 °C (16 h light/8 h dark) for 1 month. The tissue culture seedlings were then transferred to MS medium containing 1.0 mg/L 6-BA (biosharp, Shanghai, China), 0.1 mg/L NAA (Aladdin, Shanghai, China), and 30 g/L sucrose (Aladdin, Shanghai, China). The induced protocorms (PLBs) were supplemented with 1/2 MS liquid medium containing 0.1 mg/L α-naphthylacetic acid (NAA), 0.1 g/L whey protein hydrolysate, and 30 g/L sucrose (pH 5.8), and then cultured in darkness at 25 °C for 2 months. The PLBs were cut into 0.5 cm × 0.5 cm pellets, and 7 g of the pellet was inoculated into an Erlenmeyer flask containing 40 mL of MS medium. MeJA (100 μM methyl jasmonate; Aladdin, Shanghai, China), SA (100 μM salicylic acid; Aladdin, Shanghai, China), and ABA (100 μM abscisic acid; Aladdin, Shanghai, China) were filtered through 0.22 μm filter membrane and added to the MS medium, based on previously published articles [31]. After induction with SA and MeJA, samples were harvested at 0, 2, 4, 8, 24, 48, and 72 h. The original bulbs under ABA induction were harvested at 0, 24, 48, 72, 96, and 168 h. All samples were immediately stored at − 80°C after harvesting for RNA extraction. All results were based on three biological repeats and each biological repeat had three technical replicates. The extraction of total RNA from PLBs was carried out with Plant Total RNA Isolation Kit (Sangon Biotech, Shanghai, China) using 300 mg tissue homogenized in liquid nitrogen according to the manufacturer’s protocol, which was subsequently reverse transcribed into the first DNA strand using a One Step RT-qPCR Kit (BBI Life Science, Shanghai, China).

### 4.2. Screening and Identification of the *PLP_deC* Genes

We obtained the HMM (Hidden Markov Model) configuration file for the PLP_deC domain (Pfam00282) from Pfam (http://pfam.xfam.org/). All *PLP_deC* genes were then identified in the *D. officinale* and *P. equestris* genomes using HMMER software (*E*-value = 0.001) [32]. All candidate genes were submitted to Pfam and SMART for verifying the presence of the PLP_deC domain. The sequences that did not contain the conserved domain and the redundant sequence of the repeat were removed, and finally, the *PLP_deC* genes were obtained. In Table S1 are listed the sequences of the *PLP_deC* genes of *O. sativa* and *A. thaliana* described in previous studies (add citations) and used in the present work [17].

### 4.3. Sequence Attribute Analysis and Phylogenetic Tree Construction of *PLP_deC* Genes

To analyze the sequence attributes and characteristics of the amino acids of the *PLP_deC* family members, the isoelectric points (pIs) of the obtained PLP_deC amino acid sequences were determined using online analysis with the ProtParam tool (https://web.expasy.org/protparam/) [33] and subcellular localization of 8 and 6 PLP_deC-family protein sequences in *D. officinale* and *P. equestris* by Target P 1.1 (http://www.cbs.dtu.dk/ services / Target P). Properties such as molecular weight (MW) were predicted. The obtained PLP_deC protein sequences were aligned using Clustal X [34], implemented in MEGA 5.0 [35], and a maximum likelihood (ML) phylogenetic tree was generated using IQ-TREE software [36] with 1000 bootstrap replicates. The PLP_deC genes were classified according to the phylogenetic relationships. If two different species of genes are located in the phylogenetic tree at the same node and the sequence similarity is more than 80%, we consider two of these are homologous genes [37]. The conserved motifs on the orchid sequences of PLP_deC were defined by MEME (http://meme-suite.org/) using the following parameters: maximum number of motifs = 10, number of repetitions—any, and only motifs with an *E*-value < 0.01 were retained for further analysis. The motif logos of the PLP_deC domains were generated using online MEME program (Figure S2) [38], and GSDS was used to determine the exon–intron structure (http://gsds.cbi.pku.edu.cn /) [39].

### 4.4. Calculation of Ks and Ka Values of the *PLP_deC Genes*

The protein sequences of the gene pairs were first aligned using Clustal X 2.0, and then the multiple sequence alignments of proteins and the corresponding cDNA sequences were converted to codon alignments using PAL2NAL (http://www.bork.embl.de/pal2nal/) [40]. Finally, the resulting codon alignment was used to calculate Ks and Ka using DnaSP 5.0 (http://www.ub.edu/dnasp/) [41].

### 4.5. Analysis of Cis-Acting Elements of the *PLP_deC* Genes

A 2000 bp sequence upstream of the translation start site (ATG) of each *PLP_deC* gene was obtained, and an analysis of the upstream regulatory promoter elements was performed using the online tool PlantCARE (http://bioinformatics.psb.ugent.be/webtools/plantcare/html/) [42].

### 4.6. Expression of the *D. officinale PLP_deC* Genes in Different Tissues

To obtain expression data for *D. officinale*, we searched the NCBI SRA database (PRJNA348403) for RNA-sequence data from different tissues [27] (Additional file: Table S2). The raw data were stripped of adapters and low-quality reads (and bases) and then rRNA and virus reads were filtered out by using Trimmomatic software with the default parameters. The clean reads were aligned to the *D. officinale* genome using Hisat2 [43] with the options -dta and -no-unal. The aligned outputs were converted from SAM to BAM format by using SAMtools [44]. Then, the Stringtie software was used to estimate the transcript abundances with FPKM method. The heat map of the *PLP_deC* genes expression profiles was obtained by TBtools software (https://github.com/CJ-Chen/TBtools/releases).

### 4.7. Quantitative Fluorescence Analysis of the *PLP_deC* Genes

We used the CFX96 Touch^TM^ Real-Time PCR Detection System (Singapore) to perform a quantitative fluorescence analysis of the *PLP_deC* genes in the cDNA samples from *D. officinale* protocorms with three replicates. *β*-actin [45,46,47] was used as an internal reference, the relative expression levels of genes were calculated using the 2^−ΔΔCT^ method [48], and the primers were designed using Primer Premier 5.0 software (Table S3). Each reaction contained the following: 8 μL of SYBR Premix Ex Taq II (2x), 2 μL of template cDNA, 1 μL of forward and reverse primers, and addition of ddH_2_O to a final volume of 20 μL. The reaction conditions were as follows: 95 °C for 3 min followed by 40 cycles of 95 °C for 10 s, 52 °C for 15 s, and 72 °C for 30 s.

To determine whether the differential expression was significant, the difference in the relative expression of each target gene in the different treatment groups was analyzed by Student’s *t*-test in SPSS 25.0 software [49].

## 5. Conclusions

Overall, we conducted a comprehensive analysis of *PLP_deC* genes in both *D. officinale* and *P. equestris*. Comparative analysis has shown that eight *PLP_deC* genes from *D. officinale* could be divided into three subfamilies: GAD, HDC, and AAD. Most genes have an acidic pI. Purifying selection may have played a key role in the evolution of this *PLP_deC* genes in *D. officinale* based on the Ka/Ks value. Among them, both *DoAAD1* and *DoAAD2* were highly expressed in the column, flower bud, and lip. Under three hormone treatments, MeJA, ABA, and SA, the *PLP_deC* genes responded to abiotic stresses. These results provide preliminary biological information for further studies of the evolution of *PLP_deC* genes in Orchidaceae.

## Figures and Tables

**Table 1:** Sequence analysis of pyridoxal phosphate-dependent decarboxylase (*PLP_deC*) family members.

Gene Name	Gene ID	Location	pI	MW (kDa)	Pyridoxal_deC Domain
*PeGAD1*	PAXXG012610	scaffold3	5.53	55.66	√
*PeAAD1*	PAXXG064070	scaffold28	6.43	54.31	√
*PeAAD2*	PAXXG110580	scaffold67	6.68	55.69	√
*PeGAD2*	PAXXG162190	scaffold130	5.96	56.84	√
*PeGAD3*	PAXXG162200	scaffold130	8.55	57.26	√
*PeHDC1*	PAXXG233810	scaffold291	5.96	53.44	√
*DoAAD2*	*Dendrobium*_GLEAN_10136748	scaffold166	6.32	79.29	√
*DoGAD3*	*Dendrobium*_GLEAN_10126118	scaffold506	5.88	68.10	√
*DoAAD3*	*Dendrobium*_GLEAN_10085660	scaffold2906	5.83	62.7	√
*DoGAD4*	*Dendrobium*_GLEAN_10051783	scaffold6549	6.28	34.48	√
*DoGAD2*	*Dendrobium*_GLEAN_10051784	scaffold6549	6.2	51.98	√
*DoAAD1*	*Dendrobium*_GLEAN_10046548	scaffold7413	7.52	42.84	√
*DoHDC1*	*Dendrobium*_GLEAN_10045094	scaffold7529	5.67	73.82	√
*DoGAD1*	*Dendrobium*_GLEAN_10044649	scaffold7738	5.27	47.84	√

**Note:** √ shows conserved domain of Pyridoxal_deC.

**Table 2:** Subcellular localization of PLP_deC proteins.

Gene Name	Chloroplast Transit Peptide	Mitochondrial Targeting Peptide	Signal Peptide	Other	Location	Reliability Class
*DoAAD1*	0.040	0.026	0.735	0.346	S	4
*DoAAD2*	0.093	0.239	0.029	0.806	*	3
*DoAAD3*	0.039	0.109	0.561	0.525	S	5
*DoGAD1*	0.218	0.279	0.203	0.148	*	5
*DoGAD2*	0.135	0.068	0.180	0.727	*	3
*DoGAD3*	0.570	0.190	0.106	0.161	*	4
*DoGAD4*	0.015	0.261	0.229	0.061	*	5
*DoHDC1*	0.096	0.194	0.134	0.846	_	2
*PeAAD1*	0.038	0.201	0.091	0.893	_	2
*PeAAD2*	0.128	0.098	0.088	0.900	_	2
*PeGAD1*	0.324	0.135	0.110	0.383	*	5
*PeGAD2*	0.460	0.188	0.134	0.243	*	4
*PeGAD3*	0.283	0.595	0.056	0.072	*	4
*PeHDC1*	0.064	0.116	0.219	0.845	_	2

Note: Confidence levels range from 1 to 5, and 1 shows the highest predicted reliability with difference > 0.8; 2: 0.8 > difference > 0.6; 3: 0.6 > difference > 0.4; 4: 0.4 > difference > 0.2; 5: 0.2 > difference. Location: C, M, and S, represent chloroplast, mitochondrial and extracellular, respectively. _ other location, * unknown location.

**Table 3:** The Ka, Ks, and Ka/Ks values of gene pairs.

Gene Pair		Ka	Ks	Ka/Ks
*PeGAD1*	*DoGAD1*	0.6314	0.8256	0.7645
*DoGAD2*	*DoGAD4*	0.194	0.37	0.5243
*PeAAD1*	*DoAAD3*	0.3143	0.5474	0.5742
*DoAAD2*	*PeAAD2*	0.1117	0.76	0.147
*PeHDC1*	*DoHDC1*	0.1642	0.5726	0.2868
*PeGAD2*	*PeGAD3*	0.2015	0.1384	1.4559

## Supplementary Materials

Supplementary materials can be found at https://www.mdpi.com/1422-0067/21/1/54/s1.
